# Mortalidade por Doença Isquêmica do Coração no Brasil – Disparidades no Nordeste

**DOI:** 10.36660/abc.20210419

**Published:** 2021-07-15

**Authors:** Denise da Silva Pinheiro, Paulo Cesar B. Veiga Jardim

**Affiliations:** 1 Universidade Federal de GoiásInstituto de Ciências BiológicasGoiâniaGOBrasilUniversidade Federal de Goiás - Instituto de Ciências Biológicas, Goiânia, GO - Brasil; 2 Universidade Federal de GoiásGoiâniaGOBrasilUniversidade Federal de Goiás – Cardiologia, Goiânia, GO - Brasil

**Keywords:** Isquemia Miocárdica, Mortalidade, Doenças Cardiovasculares, Brasil

As doenças cardiovasculares (DCV) emergiram como um problema global de saúde, responsáveis por mais de 17 milhões de mortes anualmente, configurando a principal causa de óbitos desde a década de 60. Em especial, as doenças isquêmicas do coração (DIC) constituem a DCV mais prevalente, ocupando a primeira posição como causa de morte em todo o mundo, considerando dados até 2019. Foram causadoras de 8,9 milhões de óbitos em 2019 (16% de todas as causas), sendo associadas a um incremento de mais de 2 milhões de desfechos fatais nas duas últimas décadas.^1^

No Brasil, tem sido empreendido um esforço no sentido de disponibilizar estatísticas mais abrangentes do panorama nacional das DCV com a produção de um relatório anual^2^ que inclui estatísticas oficiais do Ministério da Saúde e dados de estudos epidemiológicos, inclusive do Estudo *Global Burden of Disease *(GBD), entre outras fontes. Foi verificada uma taxa de 83 mortes por DIC por 100 mil habitantes em 2017, sendo a DIC a principal causa de morte em todas as unidade federativas brasileiras, diferentemente de 1990, quando o acidente vascular encefálico ainda liderava em alguns estados da região nordeste.^3^

Sendo um país de dimensões continentais e marcado pelas desigualdades sociais, no Brasil, ressalta-se a importância da realização de investigações populacionais que contemplem as grandes disparidades regionais, principalmente com enfoque em regiões menos desenvolvidas (norte, nordeste e centro-oeste), de forma a estabelecer prioridades para intervenções em saúde pública.^4,5^ Nesse sentido, o artigo de Santana et al.^6^ avaliou o perfil sociodemográfico e o comportamento temporal da mortalidade por DIC na região nordeste do Brasil no período de 1996 a 2016.

Com relação a evolução temporal da taxa de mortalidade, o referido estudo verificou um crescimento significativo da mortalidade por DIC em todos os nove estados do nordeste brasileiro, embora com diferenças entre os estados, destacando-se Maranhão e Piauí, onde foram observados os maiores percentuais de mortalidade. Este resultado salienta claramente a disparidade em relação ao que é observado em regiões mais desenvolvidas no Brasil, como o sul e o sudeste, onde se verifica uma tendência de diminuição desse índice, enquanto nas regiões norte e centro-oeste observa-se uma propensão de estabilização.^7^

Essa diferença na inclinação da mortalidade por DIC entre as regiões brasileiras, com maior desfavorecimento da região nordeste, já era observada em estudo envolvendo dados de mais de duas décadas atrás - 1981 a 2001^8^. A heterogeneidade persistiu em análises subsequentes, embora tenha sido observada uma convergência das tendências nas regiões^7,9^, refletindo o peso do desenvolvimento socioeconômico e o acesso aos serviços de saúde no controle da mortalidade por DIC e outras DCV. Grave e preocupante é o fato de que o problema ainda permanece e impacta fortemente a região nordeste, conforme verificado por Santana et al.^6^

O referido autor discutiu a importância de políticas públicas, em especial na Atenção Primária à Saúde, com destaque para a relevância do Programa de Saúde da Família e, nesse contexto, concluiu que as maiores mortalidades verificadas, particularmente nos estados do Maranhão e Piauí, poderiam refletir particularidades locais e condições de vida da população, marcada pela baixa escolaridade e menor PIB *per capita*.^6^

Deve-se destacar também, como um achado importante do estudo^6^, e que merece atenção, a maior taxa de incremento anual da mortalidade por DIC na faixa etária de adolescentes. Este ponto ainda carece maior elucidação científica, devendo-se, no entanto, ponderar o provável efeito do crescimento do sobrepeso, da obesidade e da dislipidemia nessa faixa etária, que já se fazem presentes de modo significativo na região nordeste.^10-13^

Dada a diferença de desenvolvimento entre as regiões e entes da federação brasileira, que se refletem em disparidades também na saúde, reforça-se a necessidade de intensificação de políticas públicas de prevenção no nordeste. Estas passam por medidas educativas para controle dos principais fatores de risco às DIC: hipertensão, dislipidemia, diabetes, tabagismo, um forte estímulo à alimentação saudável para manutenção do peso adequado, além da melhoria do acesso e qualidade dos serviços de saúde, para se conseguir reverter a tendência da mortalidade por DIC e outras DCV nessa região.
