# Supplementary material for: Mortalidade por Doença Isquêmica do Coração no Brasil – Disparidades no Nordeste
Source: Arq Bras Cardiol. 2021 Jul 15;117(1):61–2. [Article in Portuguese] doi: 10.36660/abc.20210419 (PMC8294742; doi:10.36660/abc.20210419)
Supplement: Translation

# Translation: Mortality Due to Ischemic Heart Disease in Brazil – Northeast Disparities

**Authors:** Denise da Silva Pinheiro, Paulo Cesar B. Veiga Jardim

**Affiliations:** 1 BrasilUniversidade Federal de Goiás - Instituto de Ciências Biológicas, Goiânia, GO - Brasil; 2 BrasilUniversidade Federal de Goiás – Cardiologia, Goiânia, GO - Brasil

**Keywords:** Myocardial Ischemia, Mortality, Cardiovascular Diseases, Brazil

Cardiovascular Diseases (CVD) have emerged as a global health concern responsible for more than 17 million deaths annually, representing the main cause of death since the 1960s. In particular, Ischemic Heart Diseases (IHD) constitute the most prevalent CVD, occupying the position of leading cause of death worldwide considering data until 2019, having been responsible for 8.9 million deaths in 2019 (16% of all causes of death) associated with an increase of more than 2 million deaths in the past two decades.^1^

In Brazil, it has been undertaken an effort to provide more comprehensive statistics of the national panorama of CVD, with the production of an annual report 2 which includes the Ministry of Health official statistics and data from epidemiological studies, including the Global Burden of Disease Study (GBD), among other sources. It was verified a rate of 83 deaths due to IHD per 100 thousand inhabitants in 2017,3 with IHD being the main cause of death in all Brazilian states that year, differently from 1990, when stroke was still leading in some states in the northeast region.^3^

Being a country of continental dimensions and marked by social inequalities, in Brazil, the importance of conducting population investigations that address the great regional disparities is emphasized, mainly focusing on less developed regions (north, northeast and center-west), in order to establish priorities for public health interventions.^4,5^ In this sense, the article by Santana et al.^6^ evaluated the the socio-demographic profile and the temporal behavior of IHD mortality in northeastern Brazil from 1996 to 2016.

Regarding the temporal evolution of the mortality rate, the study found a significant increase in IHD mortality in all nine states in the northeast, although with inequalities in rates among the states, especially Maranhão and Piauí, where the highest mortality percentages were observed. This result clearly highlights the disparity in relation to what is observed in more developed regions in Brazil, such as the south and southeast, where there is a decreasing trend in that index, while in the north and midwest regions there is a tendency towards stabilization.^7^

This difference in the mortality slope due to IHD among Brazilian regions, with greater disadvantage in the northeast region, was already observed in a study involving data from more than two decades ago - 1981 to 2001.^8^ This heterogeneity persisted in subsequent analyzes, although there has been a convergence of trends among regions,^7,9^ reflecting the weight of socioeconomic development and access to health services in controlling mortality from IHD, and other CVDs. Serious and worrying is the fact that the problem still remains and strongly impacts the northeast region, as verified by Santana et al.^6^

The author discussed the importance of public policies, especially in Primary Health Care, with emphasis on the relevance of the Family Health Program and, in this context, concluded that the higher mortalities verified, particularly in the states of Maranhão and Piauí, could reflect local particularities and the living conditions of the population, which is marked by low education and lower GDP *per capita*.

It should also be noted, as an important finding in the study^6^, and that deserves attention, the highest annual increase of IHD mortality in the age group of adolescents. This point still needs further scientific elucidation, however, it should be considered the likely effect of the growth of overweight, obesity and dyslipidemia in this age group, which is already present in a significant way in the northeast region.^10-13^

Given the difference of development among Brazilian regions and states, which reflects on health disparities, the need to intensify public prevention policies in the northeast is reinforced. These undergo educational measures to control the main risk factors for IHD: hypertension, dyslipidemia, diabetes, smoking, a strong stimulus to healthy eating to maintain adequate weight, in addition to improving access and quality of health services, to reverse the trend in IHD mortality and other CVDs in this region.
